# Ambient noise levels and detection threshold in Norway

**DOI:** 10.1007/s10950-016-9566-8

**Published:** 2016-03-12

**Authors:** Andrea Demuth, Lars Ottemöller, Henk Keers

**Affiliations:** Department of Earth Science, University of Bergen, Allégaten 41, N-5007 Bergen, Norway

**Keywords:** Seismic ambient noise, Detection threshold, Norway, Seismic network

## Abstract

Ambient seismic noise is caused by a number of sources in specific frequency bands. The quantification of ambient noise makes it possible to evaluate station and network performance. We evaluate noise levels in Norway from the 2013 data set of the Norwegian National Seismic Network as well as two temporary deployments. Apart from the station performance, we studied the geographical and temporal variations, and developed a local noise model for Norway. The microseism peaks related to the ocean are significant in Norway. We, therefore, investigated the relationship between oceanic weather conditions and noise levels. We find a correlation of low-frequency noise (0.125–0.25 Hz) with wave heights up to 900 km offshore. High (2–10 Hz) and intermediate (0.5–5 Hz) frequency noise correlates only up to 450 km offshore with wave heights. From a geographic perspective, stations in southern Norway show lower noise levels for low frequencies due to a larger distance to the dominant noise sources in the North Atlantic. Finally, we studied the influence of high-frequency noise levels on earthquake detectability and found that a noise level increase of 10 dB decreases the detectability by 0.5 magnitude units. This method provides a practical way to consider noise variations in detection maps.

**Electronic supplementary material:**

The online version of this article (doi:10.1007/s10950-016-9566-8) contains supplementary material, which is available to authorized users.

## Introduction

Quantification of spatial and temporal variations of seismic noise is important for many aspects of seismology. For example, the ability of a seismic network to detect earthquakes depends on the noise levels at each individual station. Moreover, seismic noise can also be used as signal to evaluate the performance of seismic equipment and vault construction (e.g., de la Torre and Sheehan 2005; Wilson et al. 2002) and it has been used to directly investigate Earth’s structure (e.g., Shapiro and Campillo 2004; Sabra et al. 2005). A thorough investigation of seismic noise including quantification of spatial and temporal variations is therefore important.

The most common procedure to compute seismic noise was established by Peterson (1993), who developed a global noise model which is now used as global reference. He defined a new upper (NHNM) and lower (NLNM) noise level boundary in the period range 10 ^−1^–10 ^5^ s. The approach to present seismic noise was extended by McNamara and Buland (2004) who use the whole seismic record, instead of isolating quiet periods, and compute probabilities. This makes it possible to present the distribution of noise levels for the entire frequency range over long time periods.

Seismic noise can be caused by human activities, wind, and water waves. Each source generates noise in specific frequency bands. Cultural activity is the main source for increased noise at high frequencies (1–20 Hz), often resulting in strong diurnal variations (e.g., Rastin et al. 2012). Small local earthquakes fall into this frequency band, which means that the cultural noise affects their detectability. Other sources for noise at high frequencies are wind and running water (McNamara and Buland 2004). The seismic noise at intermediate periods of 4–16 s is related to ocean waves (Longuet-Higgins 1950; Hasselmann 1963). In this period range, there are two distinct peaks (McNamara and Buland 2004). The double-frequency peak (periods 4–8 s) is generated by standing gravity waves resulting from superposition of oceanic waves travelling at equal periods in opposite directions. The single-frequency peak (periods 10–16 s) is generated in coastal waters. The vertical pressure variations or *interaction of waves with the shallow sea floor is directly converted into seismic energy* (Hasselmann 1963). While these two peaks are identified on most seismic stations, their amplitudes depend on the distance to the main source area. Pierson and Moskowitz (1964) showed that the peak of the oceanic wave spectrum depends on the maximum wind speed and the length of ocean acted on by the wind. The frequencies of the peaks can be shifted slightly depending on bathymetry and dominant ocean wave period (Marzorati and Bindi 2006).

Various methods exist to quantify earthquake detection thresholds. A common approach is based on the determination of the magnitude of completeness from earthquake catalogues (e.g., Woessner and Wiemer 2005). However, as D’Alessandro et al. (2011a) pointed out, the magnitude of completeness provides no information about spatial distribution of the detection threshold. They therefore propose a more complex evaluation method, SNES, which determines location errors and spatial distribution of earthquake detections. In addition to this, Ringdal (1989) and Kværna and Ringdal (1999) consider the variability of detection thresholds over time. Their continuous threshold monitoring technique provides a way to assess non-detected events, e.g., during the coda of large earthquakes. Schorlemmer and Woessner (2008) determine a detection probability, based on magnitudes and hypocentres of past earthquakes, whereas Marzorati and Bindi (2006) compare average noise levels with synthetic spectra to derive a spatial variability in the detection threshold.

Our main objective in this study is the quantification of ambient seismic noise levels in Norway as well as their temporal and spatial variation. A second objective is to investigate the quantitative relationship between wave height and intermediate period noise levels. Finally, we look at the effect of cultural noise on detection levels using the Norwegian National Seismic Network as an example.

## Data and noise computation

**Fig. 1:**
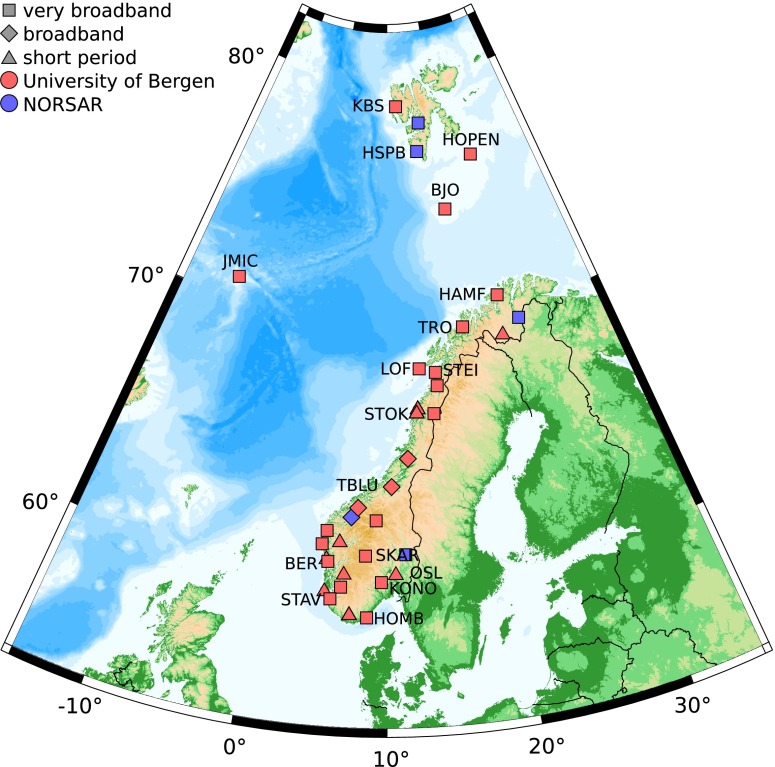
Map of the Norwegian National Seismic Network. Station codes are only given for stations that are discussed in the text. Very broadband sensors have a natural period of more than 100 s, broadband sensors of 10–60 s, and short period sensors have a natural period of less than 10 s

We evaluate the ambient seismic noise in Norway based on data recorded by the permanent Norwegian National Seismic Network (NNSN) (Fig. 1 and Table 1) as well as two temporary deployments, MAGNUS and NEONOR2. The NNSN consists of 33 stations that are run by the University of Bergen (UoB) and also includes data provided by NORSAR from three seismic arrays and two single seismometer stations. The stations are distributed over mainland Norway, as well as the arctic islands Svalbard, Bear Island, Hopen, and Jan Mayen. All stations are located on bedrock.

**Table 1:** Basic information about the NNSN stations

Station	Latitude [ ^∘^N]	Longitude [ ^∘^E]	Location	Vault
AKN	62.18	6.99	Rural, mountain slope	Shallow
ARE0	69.53	25.51	Rural	Deep
ASK	60.47	5.20	Rural	Shallow
BER	60.38	5.33	City	Vault in basement
BJO	74.50	18.99	Arctic island	Shallow
BLS5	59.42	6.45	Rural	Shallow
DOMB	62.07	9.11	Rural	Shallow
FOO	61.59	5.04	Small town	Shallow
HAMF	70.64	23.68	City	Shallow
HOMB	58.27	8.50	City	Shallow
HSPB	77.00	15.53	City	Surface
HOPEN	76.50	25.01	Arctic island	Shallow
HYA	61.16	6.18	Rural	Shallow
JMIC	70.92	-8.73	Arctic island	Deep broadband vault
KBS	78.91	11.91	Arctic island	GSN, deep
KMY	59.20	5.24	Rural	Shallow
KONO	59.64	9.59	Mine tunnel	Very deep
KONS	66.49	13.11	Rural	Shallow
KTK1	69.01	23.23	Rural	Shallow
LOF	68.13	13.54	Rural	Shallow
MOL	62.56	7.54	Rural	Shallow
MOR8	66.28	14.73	Rural	Shallow
NC602	60.74	11.54	Rural	Deep
NSS	64.53	11.96	Rural	Shallow
ODD1	59.91	6.62	Rural	Shallow
OSL	59.93	10.72	City	Vault in basement
SKAR	60.68	8.30	Rural	Deep broadband vault
SNART	58.33	7.20	Rural	Shallow
SPA0	78.18	16.37	Arctic island	Borehole
STAV	58.93	5.70	City	Basement
STEI	67.93	15.24	Rural	Shallow
STOK	66.33	13.01	Rural	Shallow
SUE	61.05	4.76	Rural	Shallow
TBLU	63.41	10.43	City	Basement
TRO	69.63	18.90	City	Museum

The permanent stations are operated by two institutions with different aims, and, therefore the site and vault conditions differ. The majority of the stations operated in Norway were installed in the 1980s and 1990s for use with short period seismometers. The vaults constructed then were shallow, less than 1 m below the surface, but coupled to bedrock. At many of these sites, the short period instruments have been replaced by broadband seismometers, but the vaults remained the same. In 2013, 21 of the seismic stations were equipped with broadband seismometers, mainly Nanometrics Trillium 120 seismometer, and recording was done with Güralp CMG-DM24 digitizers. A deeper vault of about 2 m at station SKAR was built more recently for a broadband sensor. The Svalbard array is equipped with 10-m-deep borehole sensors. The two stations KBS and KONO are part of the Global Seismograph Network (GSN) that have been constructed to produce high-quality data and can thus be used as a reference. KBS on Svalbard has a well constructed GSN style vault, while KONO located in southeastern Norway is placed in a tunnel of an abandoned silver mine.

Stations of the MAGNUS and NEONOR2 deployment were placed in existing buildings. During the MAGNUS project, a total of 31 stations recorded in southern Norway for 2 years (2006–2008; Weidle et al. 2010). They used 23 Streckeisen STS2 sensors, 6 Geotech KS2000, and 2 Güralp 40T. The NEONOR2 project deployed a total of 26 stations, 5 Trillium 120, 15 Streckeisen STS2.5, and 6 Güralp 3ESP sensors in northern Norway in 2013. These stations are scheduled to record until April 2016.

When evaluating the noise levels, we have to consider the different installation techniques as, in particular at long periods and for horizontal components, the noise is sensitive to the vault construction (e.g., Vassallo et al. 2012). Shallow vaults, bad insulation, and air circulation also increase long period noise (Díaz et al. 2010; Vassallo et al. 2012; Bormann 2012). Our noise analysis is based on data recorded in 2013. We mainly focus on the NNSN stations, but the analysis of the noise model of Norway includes the temporary networks as well.

We computed noise levels in terms of power spectral density (PSD) with the noise computation implemented in SEISAN (program CONNOI (Ottemöller et al. 2010)), which follows McNamara and Buland (2004). Noise levels are computed for equally spaced log(f) values, where interpolation is applied if required. Otherwise, no smoothing across frequencies is applied. We used no overlap for spectrograms and a window length of 15 and 60 min for diurnal and seasonal variations, respectively. Probability density functions (PDFs) are calculated using a 60 min window and 50 % overlap. We calculated the noise levels for all three components. However, for our results, we always use the vertical component.

The PSDs are calculated in decibels with respect to acceleration of 1 $(\frac {\mathrm {m}}{\mathrm {s}^{2}})^{2}/\text {Hz} $ using:

1 $$ P_{k}=\frac{2{\Delta} t}{N} |Y_{k}|^{2}  $$

The total power *P*_*k*_ is proportional to the square of the amplitude spectra |*Y*_*k*_|. In order to compare the PSD with Peterson (1993), the normalization factor of twice the ratio of the sample interval Δ*t* to the number of samples *N* is needed. Furthermore, we applied a correction factor of ∼1.143 to account for the used 10 % taper. To analyze the statistical noise variation over a certain time period, we computed PDFs using:

2 $$ P(T_{c})=N_{PT_{c}}/N_{T_{c}}  $$

Here, *P*(*T*_*c*_) is the probability for a given center period *T*_*c*_, $N_{PT_{c}}$ is the number of spectral estimates that fall into a 1-dB power bin, and $N_{T_{c}}$ is the total number of spectral estimates. The mode values of the PDFs were averaged over the frequency ranges 2–10 Hz, 0.5–5 Hz, and 0.125–0.25 Hz. These ranges represent, respectively, the frequencies where the highest signal energy of small local and regional events, teleseismic events, and the double-frequency microseism peak is expected.

## Temporal noise variation

In this section, we present and evaluate the diurnal and seasonal variations in the seismic noise. Changes in cultural activity are expected to be visible for higher frequencies between day and night. Variations due to seasonal weather changes are expected to be seen at lower frequencies, especially the microseism peaks. All provided times are in UTC and local time. The UTC is in the winter 1 h and in the summer 2 h behind local time.

### Diurnal variations

**Fig. 2:**
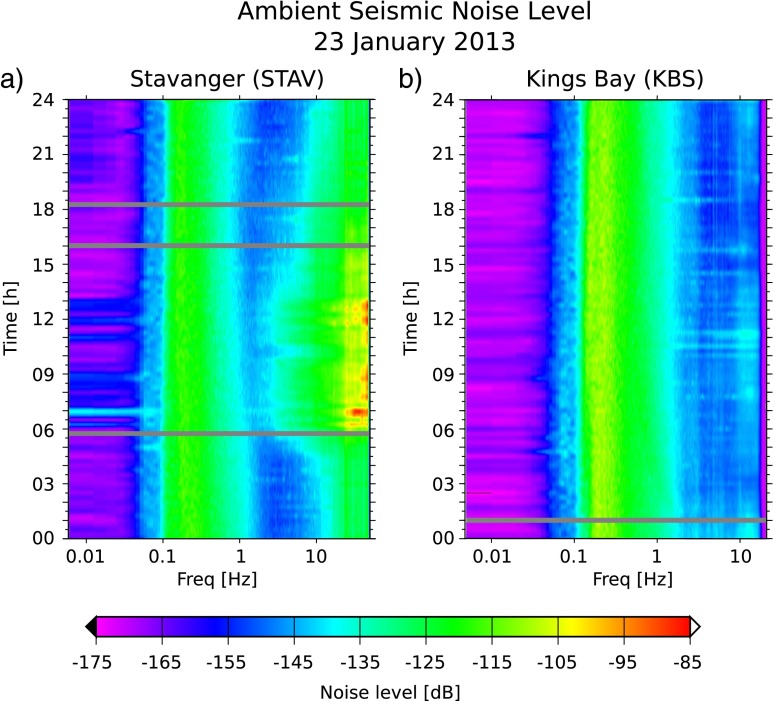
Twenty-four-hour PSD spectrogram of the vertical component for **a** STAV and **b** KBS on the 23 January 2013. Time is given in UTC, which is 1 h behind local time

As an example of the difference between a culturally quiet and noisy station, we show in Fig. 2 the 24-h PSD spectrograms of stations KBS and STAV. KBS is installed on the arctic archipelago Svalbard about 1 km from the coast near a small settlement. The station in Stavanger is placed in the basement of a building in an industrial area. Figure 2a shows that the noise levels at STAV for frequencies above 4 Hz increase at 5 a.m. (6 a.m. local time) and decrease again around 4 p.m. (5 p.m. local time), correlating with the daily working hours. KBS (Fig. 2b), on the other hand, shows no obvious variation in this frequency range due to the absence of cultural activity. Most NNSN stations show a diurnal variation of less than 5 dB (Online Resource 1, Table ??1). However, stations located in larger towns, e.g., Stavanger, Trondheim, and Bergen, show variations of up to 15 dB. Furthermore, the daytime cultural noise for these stations is greater during working days than in the weekend.

McNamara and Buland (2004) observed noise variations of 15–20 dB for higher frequencies (1–100 Hz) across the USA. Their lowest diurnal variation was 10 dB. Marzorati and Bindi (2006), who studied noise in northern Italy, observed diurnal variations for frequencies higher than 1 Hz between 10 and 20 dB for different sites. Similar variations were observed by Rastin et al. (2012) for the North Island of New Zealand for a frequency range of 1–10 Hz (diurnal variations: 7–20 dB). Compared to those studies, the NNSN sites in Norway show less diurnal variation. This is partly explained by the sparse population density in Norway compared to the other countries.

The comparison of diurnal variations between January and July (Online Resource 1, Table ??1) shows a significantly lower variation in July for the southern sites with a maximum of 8 dB. On the other hand, the northern stations have an increased diurnal variation in July. As an extreme example, Hammerfest has a diurnal noise level variation of 4 dB during January and 10 dB during July. Hence, we observe an increase in the cultural activities in northern Norway only. This is similar to observations by Rastin et al. (2012) in New Zealand for the summer months. Explanations for those observations could be snow coverage and summer holidays in Norway. July is the month of school summer holidays, which reduces regular daily traffic in the southern cities. The snow coverage in northern Norway is a possible reason for noise attenuation during the winter, thus smaller noise levels.

### Seasonal variations

**Fig. 3:**
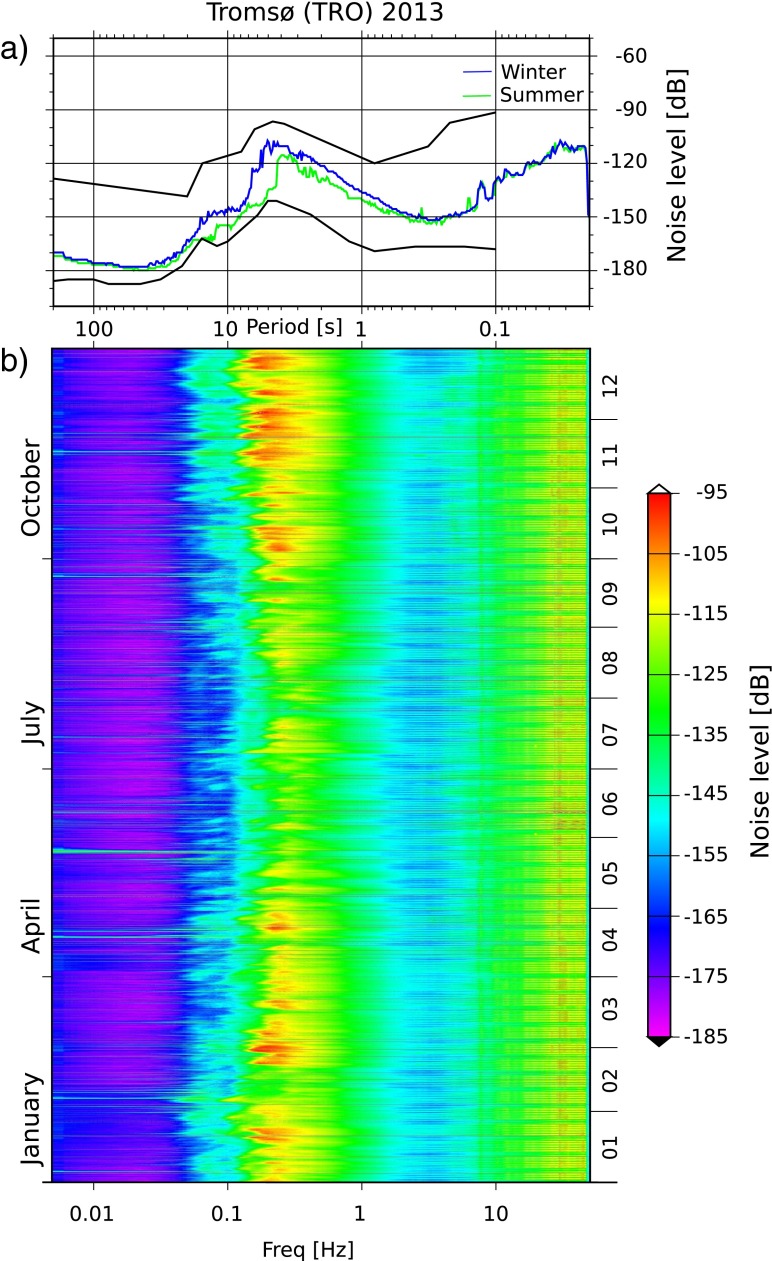
**a** PDF mode values for winter and summer 2013. The *solid black lines* show the NHNM and NLNM of Peterson (1993), respectively. **b** PSD spectrogram of 2013. Both plots were calculated for the vertical component of station TRO

Seasonal variations in noise levels are caused by seasonal changes in the weather (e.g., Stutzmann et al. 2000; Traer et al. 2012) and also depend on the offshore bathymetry (e.g., Longuet-Higgins 1950; Kedar et al. 2008). In order to analyze the seasonal noise variations in Norway, we chose 13 stations, representative for island, coastal, and inland stations (Online Resource 1, Table ??2). Figure 3 shows an example of the PSD and the PDF mode values for the summer and winter months for TRO. This station is installed in the basement of a museum in Tromsø.

The most significant noise level variation in the PSD occurs for periods between 1 and 35 s. The noise levels are high in the period October–March (winter) and low in the period April–September (summer). During the summer months, the noise levels stay low, apart from a few individual peaks related to individual storms. The summer and winter modes show that the microseism peaks have different amplitudes and occur at different frequencies, with the peaks shifted by 1–2 s toward shorter periods in the summer. These variations result from rougher weather conditions with longer ocean wave periods during winter (e.g., Bretschneider 1959).

The highest seasonal variation, 7–22 dB, in Norway is observed for low frequencies (0.125–0.25 Hz). This compares to 25 dB reported by Marzorati and Bindi (2006) for a frequency band of 0.1–0.3 Hz in Italy, 15–20 dB (f ∼0.125 Hz) by McNamara and Buland (2004) for the USA, 20 dB (f ∼0.111 Hz) by Díaz et al. (2010) for Iberia and Morocco and 6–10 dB (*f* = 0.25–1 Hz) by Rastin et al. (2012) for New Zealand.

The noise levels in the winter for the frequency range 0.5–5 Hz are slightly higher (0.24–11.58 dB) than during the summer (Online Resource 1, Table ??2). In the frequency range 2–10 Hz, we observe that half of the analyzed stations have a higher noise level during the summer (0.48–4.88 dB) and the other half during the winter (0.12–19.4 dB). However, the stations with increased noise levels show no geographical pattern. This therefore implies a weather-independent seasonal noise level variation for this frequency range.

## Weather conditions and ambient seismic noise

In this section, we study the link between ocean waves that result from the weather conditions and the seismic noise levels in northern Norway.

Wind speed (10 m above sea level) and wave height values offshore northern Norway for 2013 were provided by the Norwegian Meteorological Institute. The area covered is [0 ^∘^–35 ^∘^ E] and [66 ^∘^ N–75 ^∘^ N], with a grid resolution of 10 km and a 3-h time resolution. The wind speed and wave height values are based on the operational model from the European Centre for Medium-Range Weather Forecasts, which is a high resolution numerical weather prediction model (Reistad et al. 2011). The model uses temperature, pressure, wind, specific humidity, and cloud water observations for atmospheric modelling. The ocean wave field is then generated with the wave prediction model coupled to the atmospheric conditions (Reistad et al. 2011).

From these data, we calculated average wind speed and wave height values, as well as local maxima. This was done in 50-km intervals around the corresponding station with a distance increment of 10 km and an azimuth increment of 1^∘^. Given the wave height *w*(*r*,*𝜃*), with *r* distance of the wave to the station and *𝜃* the azimuth, we compute the average wave height for each bin using:

3 $$ \overline{w} = \frac{\int\limits_{\theta_{1}}^{\theta_{2}}\int\limits_{r_{1}}^{r_{2}} w(r,\theta)r dr d\theta}{ \int \limits_{\theta_{1}}^{\theta_{2}}\int\limits_{r_{1}}^{r_{2}} r dr d\theta}  $$

**Fig. 4:**
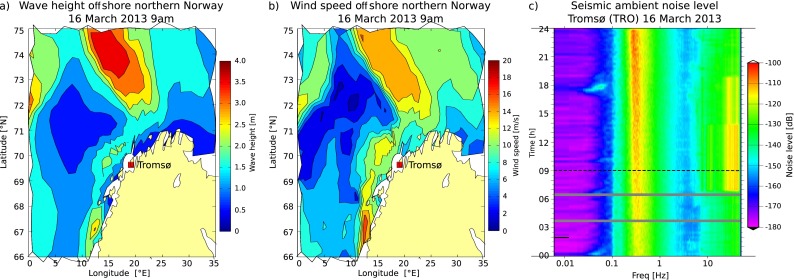
Modeled **a** wave height and **b** wind speed offshore northern Norway on March 16th 2013 at 9 a.m. Both wave height and wind speed scale are given on the y-axis to the right, where the units are m and m/s, respectively. **c** Twenty-four-hour PSD spectrogram of Tromsø vertical component for March 16th 2013

The average wind speed is computed by replacing wave height by wind speed in Eq. 3. Figure 4 shows the wave height and wind speed maps for northern Norway on March 16th 2013 at 9 a.m. This figure also shows the 24-hour PSD spectrogram at station TRO on that day. A low-pressure weather system was moving toward the coast of Norway (at 9 a.m. centered at ∼74 ^∘^ N and ∼15 ^∘^ E). The spectrogram shows that the noise level around the double-frequency peak at about 4–7 s (0.143–0.25 Hz) increases very clearly from −115 dB at 9 a.m. to −100 dB at midnight.

**Fig. 5:**
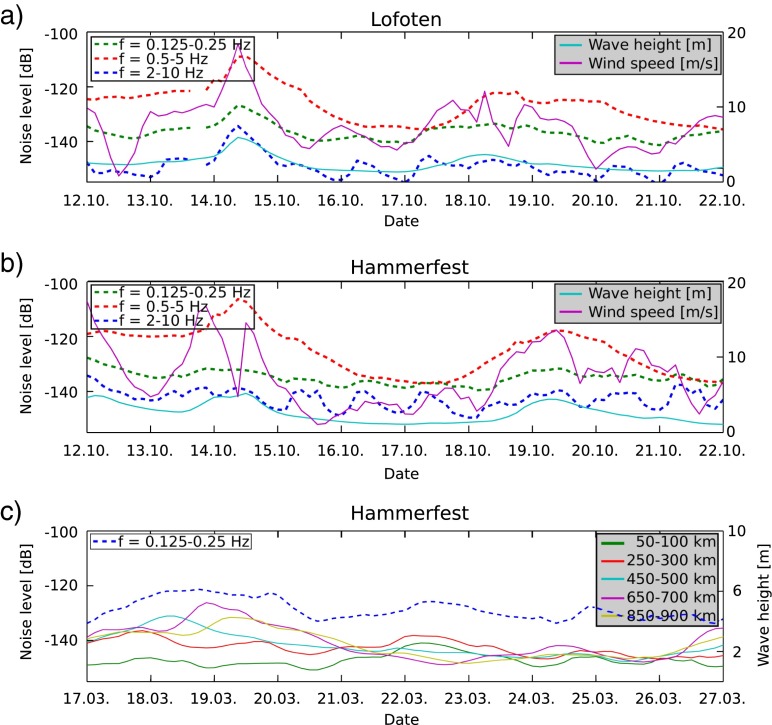
Relationship of seismic ambient noise levels of the vertical component and average wave heights at defined distances for **a** Lofoten and **b** Hammerfest in October. Wave heights are averaged over the distance of 250–300 km away from the corresponding station. Wind speeds are averaged over a distance of 50–100 km. **c** Seismic ambient noise levels of frequencies 0.125–0.25 Hz compared to average wave heights at various distance ranges in March

Ardhuin et al. (2012) suggest that noise at a single station can be related to an area averaged wave height. We investigate this by comparing average wind speeds and average wave heights at various distance ranges with the noise levels in our three frequency bands. Figure 5a, b show this comparison for average wave heights between 250 and 300 km away from the station and average wind speeds of 50–100 km distance over 10 days for the stations LOF and HAMF. We chose large offshore distances with a high correlation coefficient between noise and weather conditions. Both stations reveal a strong correlation of *r*_*LOF*_ = 0.84 and *r*_*HAMF*_ = 0.79 (Online Resource 1, Table ??3) between wave heights and noise levels in the low-frequency band (0.125–0.25 Hz). We also find that the peak in time for higher frequencies correlates with the wave height peak.

For station LOF, which has a shallow vault with a depth of less than 1 m, the wind speed shows an expected high correlation of *r* = 0.62 (Online Resource 1, Table ??3) with the noise at high frequencies (2–10 Hz). An additional reason for the strong correlation at LOF could be its position on a peninsula. The effect of wind on the higher frequency noise depends both on the conditions around the site and the burial depth of the seismometer (e.g., Carter et al. 1991; Bormann 2012). This correlation is for example not clearly observed for station HAMF (Online Resource 1, Table ??3). Other stations near the coast show a similar relationship between average wind speeds and noise levels for frequencies 0.5–5 and 2–10 Hz. Seismic noise due to wind attenuates quickly and thus is predominantly generated near the seismic station. Carter et al. (1991) showed high-frequency noise as function of depth and found that prevalent wind generated noise at the surface. Subsurface stations recorded no noise for frequencies above 3 Hz. However, seismic noise generated in the ocean over large areas results from a combination of weather conditions (e.g., Bormann 2012).

We are also interested in evaluating the distance to the station for which wave height data have an effect on the seismic noise levels, thus can be used as a proxy. Figure 5c presents the low-frequency noise levels together with the wave heights for various distance ranges. Using data in the period March 17th–27th 2013 reveals that the link between wave height and noise is strongest when considering near-coastal wave heights. However, wave heights up to 900 km offshore can be used as a proxy to estimate noise levels closer to the coast (see Online Resource 1, Table ??4). Since wave height levels at various distances are not independent from each other, as seen in Fig. 5c, evaluation of the correlation allows no general conclusion that wave heights at larger distances influence the observed noise levels. Nevertheless, our average wave height values at 850–900 km offshore have a correlation coefficient of 0.73 (Online Resource 1, Table ??4) with noise levels for low frequencies in the year 2013. Thus, our observations agree with Ardhuin et al. (2011), who proposed that reflections within 1000 km offshore increase the seismic noise level for the double-frequency peak.

**Fig. 6:**
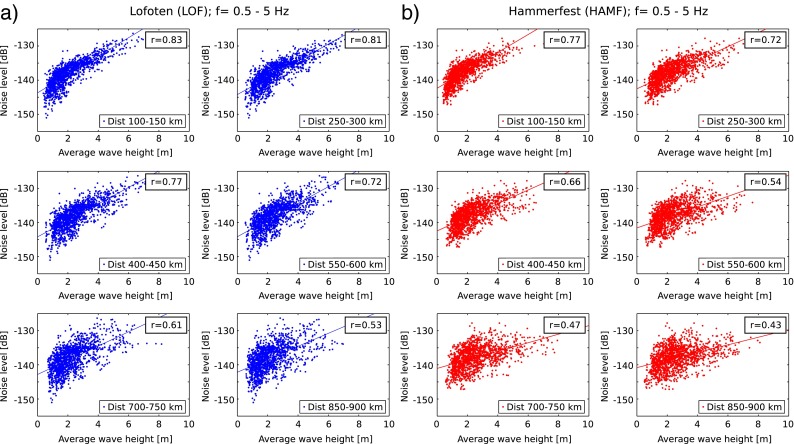
Relationship of seismic ambient noise levels of the vertical component and average wave heights for six distance ranges for **a** Lofoten and **b** Hammerfest. Correlation coefficients are shown in the upper right insets

While the link between the microseism peaks and ocean wave heights is well established (e.g., Bromirski et al. 2005; Ardhuin et al. 2012), we also tested the link in our intermediate frequency band. Figure 6 shows the noise levels at frequencies 0.5–5 Hz against the average wave heights for various distances from the station HAMF and LOF together with the corresponding correlation coefficients. For distances up to 600 km offshore, we observe a strong correlation (*r*>0.7) between increasing wave heights and increasing noise levels. With increasing distance, the correlation becomes more scattered. We also see a correlation for higher frequencies (2–10 Hz), which may be due to increased wind speeds that correlate with wave heights.

**Fig. 7:**
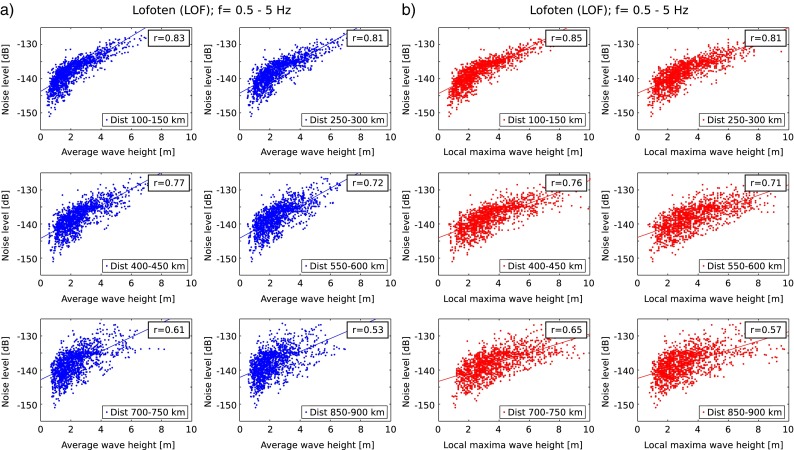
Relationship of seismic ambient noise levels of the vertical component and wave heights for six distance ranges for Lofoten. **a** Noise levels versus average wave heights. **b** Noise levels versus local maxima wave heights. Correlation coefficients are shown in the upper right insets

Instead of using wave heights averaged over a distance interval to estimate the change in noise levels, it would also be possible to use the maximum wave height within a distance range. To test which of the two is a better proxy, we made a comparison in Fig. 7. The dependency of noise levels on the local maximum wave height is slightly higher (see Fig. 7 for correlation coefficients). As the maximum is slightly easier to estimate, the local wave height maximum is a good approximation for the noise level wave height relation, even though the noise is expected to be generated over a larger area, as suggested by Ardhuin et al. (2012), for regions such as north-western Europe.

## Noise model for Norway

Another factor of importance in this study is the geographic noise variation. For frequencies around the microseism peaks, this can be related to differences in the natural ambient noise. At frequencies lower than the single frequency peak, the individual station noise mostly represents the seismic station setup (Bormann 2012). For the higher frequencies, the noise largely reflects the proximity to cultural activity. To evaluate the performance of a seismic station and to identify needs for improvement, it is crucial to develop a local noise model.

**Fig. 8:**
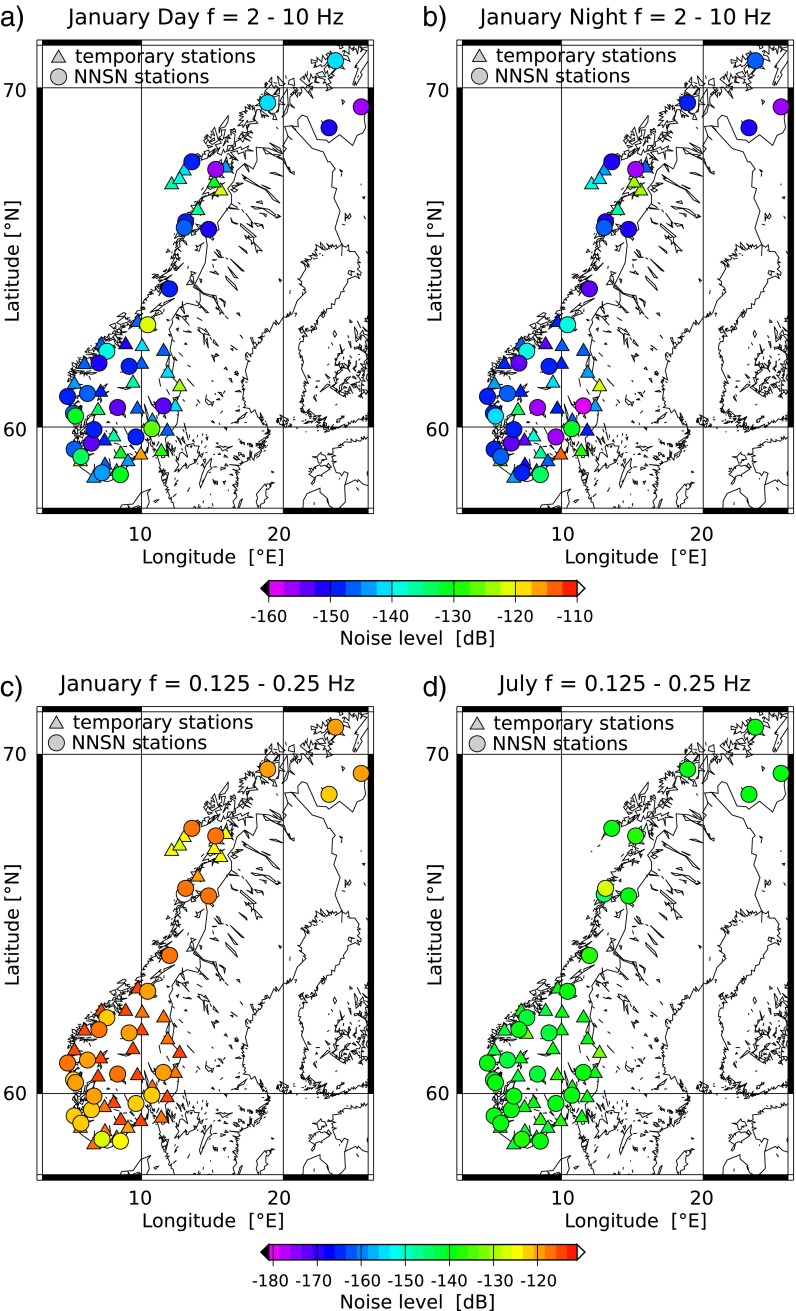
Geographic noise distribution of the vertical component in Norway. Comparison of **a** day and **b** night in January 2013 for frequencies 2–10 Hz. Noise level variations between **c** January and **d** July are shown for frequencies 0.125–0.25 Hz

In order to assess the geographic noise distribution, the average mode values of the individual stations are shown in Fig. 8. This was done for all three of our chosen frequency ranges for day and night time in January and July. Additionally, we included mode values of temporary stations from the MAGNUS project (July 2007; January 2008) and the NEONOR2 project (January 2014). The temporary stations generally show higher noise levels than the nearby permanent station. This was expected, largely due to the fact that the temporary stations are installed inside buildings. The comparison of day versus night and January versus July (Fig. 8) reflects our above-mentioned observations. We have higher noise levels in the frequency range 2–10 Hz during the day for city stations and higher noise levels in the frequency range of 0.125–0.25 Hz in January.

Figure 8c suggests that stations in southern Norway have slightly lower noise levels than the northern stations for the frequency band 0.125–0.25 Hz. This can be explained by the larger distance to the dominant noise source regions in the northern Atlantic. The noise levels hardly vary between stations in July for this frequency range (Fig. 8d). We could have expected to find that stations near the coast have higher microseism peak amplitudes, but that difference is not significant. Overall, the noise level variation in Norway shows no clear geographic pattern. This may be the result of low attenuation and an indication that the geology has no significant effect on the regional noise pattern.

**Fig. 9:**
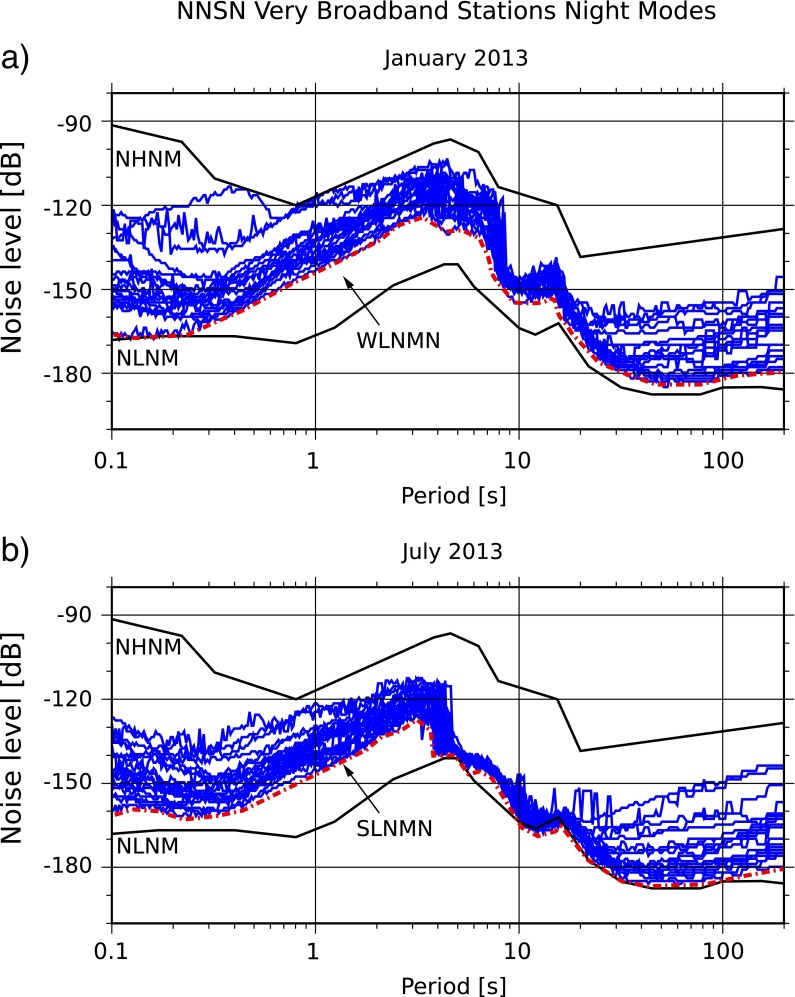
Low noise model for Norway (LNMN) constructed from the minimum mode values of the vertical component from most very broadband stations of the NNSN. The *red line* marks the lowest noise levels in Norway for night time (6 p.m.–6 a.m.) in **a** January 2013 and **b** July 2013

The best case scenario of the network performance is given by the low noise summer and winter models for Norway (SLNMN, WLNMN; Fig. 9). They are constructed from the minimum mode values of most very broadband stations. Two stations of the network had to be omitted due to technical problems in the analyzed time period.

The shape of both curves are in good agreement with each other as well as with the Peterson (1993) model for periods shorter than 1 s and longer than 20 s. We observe a shift of the single- and double-frequency peak toward longer periods in January as explained in the discussion of Fig. 3. An increased scattering between stations and higher noise levels for periods between 1 and 20 s is observed in January. This period band includes the microseism peaks. As discussed in the weather section, weather conditions are rougher during the winter months. This causes higher scattering in the observation. The local maxima around 6 s are significantly lower in July.

Various studies observe a splitting in the double-frequency peak (e.g., Stephen et al. 2003; Bromirski et al. 2005; Kuper and Burlacu 2015). Bromirski et al. (2005) distinguish between short (2–5 s) and long (5–11 s) double-frequency peaks. They assume a nearby local storm source for the short period peak and larger storms in the open ocean as sources for the longer period peak. If the same applies to Norway, the noise levels in Norway caused by local storms are more stable than the ones triggered by distant storms.

Noise levels at longer periods are partly related to the vault construction and seismometer self-noise (McNamara and Buland 2004). We find that the best constructed stations in Norway (KONO, KBS, SKAR) perform well for all their components, but for shallow vaults, the range of observations is quite large. The highest noise levels at long periods are observed for HOMB, HOPEN, and STAV. This is not surprising, since the vault and site conditions for these stations are not favorable.

To quantify the noise level variation between stations, we calculated the average mode of all mainland and island stations, respectively, and subtracted the individual mode values (Online Resource 1, Tables ??5 & ??6). This was done for the three frequency ranges. The highest variation of 26 dB between mainland stations is observed in January for the frequency range 2–10 Hz between OSL and STEI. STEI has a 10-dB lower noise level for those frequencies than the average. We observed a noise level variation of approximately 16 dB between the quietest and noisiest mainland station for the frequency range 0.5–5 Hz and 9 dB for frequencies 0.125–0.25 Hz. In July, we observed similar variations of 15 dB and 24 dB for the frequency ranges 0.5–5 Hz and 2–10 Hz. The noise level variation for the frequency range 0.125–0.25 Hz is 8 dB higher in July (17 dB) than in January.

The variance of noise levels between island and mainland stations is stable in January and July for all frequency ranges. The average noise value of island stations in the frequency range 0.125–0.25 Hz is the same as for mainland stations. For the other two frequency ranges, we observe 6–8 dB higher average noise levels at the island stations. The highest noise level variation of 38 dB between stations is observed for the frequency range 2–10 Hz. The overall noisiest stations are JMIC and HOPEN, whereas SPA0 is the quietest station. Higher noise levels, as observed for OSL and JMIC, make earthquake analysis (teleseismic, local, and regional) more difficult. Lower noise levels, as, e.g., recorded by STEI and SPA0, contribute to the detectability of smaller earthquakes.

## Effect of noise on detection threshold

So far, we focused on the characterization of ambient noise and the evaluation of seismic station performance. In this final section, we discuss the effect of noise on seismic observations.

It is quite obvious that the detection of earthquakes and the observation of seismic phases depend on the noise levels at a station. In principle, one also expects to find larger travel time residuals for seismic phases observed on noisier stations. This could not be confirmed from the NNSN earthquake catalogue. A possible reason for this is the practice of only reading phases when the signal to noise ratio is high enough. Also, the increase in arrival time error due to noise is likely to be smaller than the error caused by the velocity model that was used. A second important observable is the detection level. The detection levels for Norway were calculated based on the requirement to have four detecting stations. In this case, the detection level is given by the magnitude that can be seen by the most distant of the four stations. The magnitude–distance relationship derived from the NNSN earthquake catalogue of the past 25 years is:

4 $$ M_{L}(d)= 0.5 + 0.004 d .  $$

**Fig. 10:**
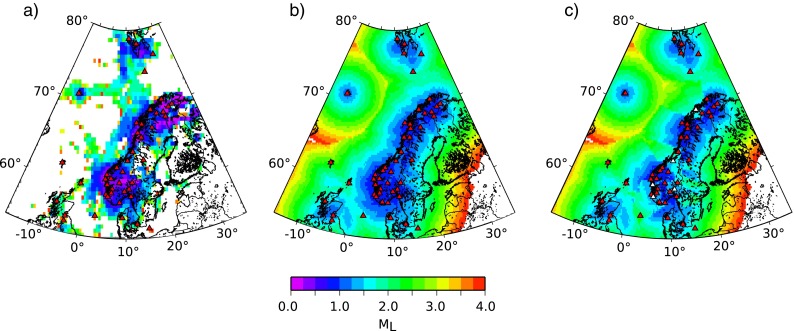
**a** Smallest local magnitude observed by the NNSN. **b** Synthetically calculated detection threshold for Norway. **c** Implementing day noise levels in the synthetic detection threshold of the vertical component for stations marked in *white*

In other words, at distance *d* (in km), a magnitude *M*_*L*_ can be detected. We computed a threshold map for Norway (Fig. 10b) by simply computing the minimum expected magnitude at the fourth nearest station for each grid point using Eq. 4. For this, we included stations from other networks, where we have data access.

Our detection levels are defined by the network geometry and the expected detection level is based on the earthquake catalogue. This is similar to Schorlemmer and Woessner (2008), who derived detection thresholds for southern California based on station locations and event magnitudes. They calculated the detection probability of an event at a certain station and used this to derive a threshold map. A more complex approach to assess the detectability of earthquakes by seismic networks was published by D’Alessandro et al. (2011a). They focused on the spatial detectability of a given magnitude and considered the spatial noise level variations, the velocity model used and the accuracy of the earthquake detection in time and space. We argue that our simple approach gives a first order estimate of detection levels and allows for easy incorporation of noise level variation between stations. Our computed detection thresholds (Fig. 10b) compare well to the smallest local magnitudes observed (Fig. 10a).

The synthetic map reveals a local magnitude detection threshold for mainland Norway of *M*_*L*_=1, whereas the detection threshold in the Norwegian Sea is as high as magnitude 3. In other places, work based on the methodology by D’Alessandro et al. (2011a) found a threshold magnitude of mainly 2–2.5 for mainland Italy (D’Alessandro et al. 2011a), 1.8 for Greece (D’Alessandro et al. 2011b), and 1.4 for Alaska (D’Alessandro and Ruppert 2012). The coastal areas in Italy and Greece have detection thresholds of a magnitude around 2.5. While this gives an indication that our numbers are comparable to other networks, network geometry, station performance, and seismic attenuation can result in significant differences.

The noise level variations determined in the previous sections are used to calculate variabilities in earthquake detection.

5 $$ {\Delta} M_{L}=log|a_{1}(t_{i})/a_{2}|  $$

For this purpose, we calculated the ratio of the average peak amplitude of a seismogram (*a*_2_) and the peak amplitude (*a*_1_) at a specific time *t*_*i*_. Equation 5 provides us with variations in magnitude detectability. The peak amplitudes *a*_1_ and *a*_2_ in Eq. 5 are derived from the power spectral values (P) over a frequency range |*f*_*S*_,*f*_*E*_| using:

6 $$ a=1.25\sqrt{P(f_{E}-f_{S})}  $$

Havskov and Alguacil (2004) and Peterson (1993). Therefore, we have a relation between the variation in local magnitude detectability and noise levels. For an increase in noise levels of 10 dB, the detectability of earthquakes decreases by a local magnitude of 0.5.

The above presented variations in noise levels this relation provide us with the following observations:

- Detection level of local and regional earthquakes in bigger cities during the day increases by up to 0.75 units of local magnitude
- Seasonal noise variation changes the detectability of teleseismic events by 0.25 units of magnitude
- Detectability of regional and local events of individual stations can vary by two units of magnitude
- Detectability of teleseismic events can vary up to 1.5 units of magnitude (e.g., an increase in the average wave height level of 4 m at a distance of 100–150 km decreases the detectability of teleseismic events at HAMF by approximately 0.5 orders of magnitude Fig. 6)

The translation of difference in noise into magnitude provides a simple way to consider noise variation in detection maps. The impact of increased noise levels on the detection threshold of Norway is shown in Fig. 10c. In that figure, we increased the detection threshold of stations with the highest day and night noise level variation (OSL, STAV, BER, TBLU, TRO) by 0.5 units of magnitude. The increase of detectability influences especially the detection threshold in the offshore areas. The higher threshold in the offshore areas caused by the higher noise levels around STAV and TBLU is reflected in the observed earthquake catalogue (Fig. 10a).

## Conclusion

We have computed the ambient seismic noise levels for Norway to investigate temporal and spatial noise variation, and to develop a local noise model. The daily noise level variations correlate with cultural activity mostly in the bigger cities. The differences in cultural seismic noise between stations were considered in the computation of detection maps, confirming that high noise levels have a significant negative effect on earthquake detection. Comparison with the smallest observed magnitudes from the earthquake catalogue shows that a fairly simple approach provides useful results that can be used to plan modifications to a seismic network. We evaluate the strong correlation between seismic noise and weather conditions for Norway. We showed in particular that local wave height maxima are a good approximation for the noise level wave height relation and that wave height variations closer to the stations have a stronger influence on the noise level. We quantified the relation between wave height and noise levels at frequencies around the double-frequency peak. No clear geographical pattern of noise level variation could be found for Norway, indicating that the ocean-generated noise propagates quite efficiently in this area. Using the mode noise levels of most very broadband stations in Norway, we constructed a low-noise model for January and July 2013. The comparison of noise levels between stations allowed us a performance evaluation of the network. Thus, monitoring of seismic noise over time provides an excellent quality control measurement.

## Electronic supplementary material

Below is the link to the electronic supplementary material.

(PDF 89.0 KB)
