# Supplementary material for: Ambient noise levels and detection threshold in Norway
Source: J Seismol. 2016 Mar 12;20(3):889–904. doi: 10.1007/s10950-016-9566-8 (PMC5270885; doi:10.1007/s10950-016-9566-8)
Supplement: Supplementary file 1 — (PDF 89.0 KB) [file 10950_2016_9566_MOESM1_ESM.pdf]

Journal of Seismology

Electronic supplementary material 1 - Tables

Ambient noise levels and detection threshold in Norway

by Andrea Demuth, Lars Ottemöller, Henk Keers

Corresponding author: A. Demuth,
Department of Earth Science, University of Bergen, Allégaten 41, N-5007 Bergen, Norway; Tel.: +47-55583661, E-mail: Andrea.Demuth@uib.no

Table 1 Diurnal noise level variation of the vertical component of mainland stations for given time and frequency ranges (f_1=0.125-0.25 Hz; f_2=0.5-5 Hz; f_3=2-10 Hz). The numbers are given in dB. Day time is considered to be between 6 am and 6 pm.

Station	January			July		
	f_1	f_2	f_3	f_1	f_2	f_3
AKN	0.54	1.04	2.62	0.12	0.82	1.78
ARE0	0.92	0.20	0.20	0.54	0.00	0.86
ASK	0.40	1.50	3.42	0.14	1.50	1.40
BER	0.22	3.96	9.24	0.60	3.22	7.36
BLS5	0.68	0.34	1.06	0.38	0.88	2.00
DOMB	0.48	0.44	1.24	1.24	1.18	3.12
HAMF	0.06	0.36	4.48	0.34	3.10	10.36
HOMB	0.26	0.18	0.08	0.18	0.38	0.56
HYA	0.26	0.80	1.62	0.38	1.70	1.16
KMY	0.14	1.40	2.70	0.56	0.08	0.64
KONO	0.54	2.34	6.70	0.76	2.68	5.76
KONS	0.12	0.46	0.70	0.66	2.84	1.42
KTK1	1.20	0.46	0.26	1.78	0.62	0.98
LOF	1.44	0.10	2.34	2.20	3.08	4.48
MOL	0.30	0.00	0.00	0.36	0.38	0.02
MOR8	0.14	0.44	1.12	2.14	1.58	2.18
NC602	0.32	1.46	4.58	0.18	2.28	4.84
NSS	0.36	2.22	4.54	1.46	3.04	4.52
ODD1	0.50	0.02	0.30	1.22	0.22	0.80
OSL	0.02	4.54	6.56	1.62	5.52	7.64
SKAR				0.10	1.26	3.58
SNART	0.86	0.78	1.90	0.08	1.16	1.78
STAV	0.62	6.92	14.50	1.06	2.44	7.10
STEI	0.94	0.24	0.98	1.06	2.00	2.18
SUE	0.10	0.04	0.22	1.02	0.98	1.62
TBLU	0.14	6.24	13.22	0.22	5.16	8.32
TRO	0.56	2.96	7.04	0.12	3.58	10.20

Table 2 Seasonal noise level variation 2013 of the vertical component for given frequency ranges (f_1=0.125-0.25 Hz; f_2=0.5-5 Hz; f_3=2-10 Hz). The numbers represent the average noise level of the whole year minus the noise level of the summer months April till September in dB.

Station	f_1	f_2	f_3
BJO	15.54	4.32	7.36
DOMB	17.52	0.46	4.02
HAMF	17.08	2.90	0.48
HOMB	6.88	1.16	0.12
HSPB	10.70	1.68	1.48
JMIC	19.60	3.28	6.80
KBS	14.22	0.88	1.76
KONO	17.44	0.90	2.74
LOF	17.10	3.32	1.66
MOR8	15.44	1.92	1.02
NSS	14.78	0.24	4.88
SKAR	18.12	2.38	1.26
SUE	21.72	11.58	19.40
TRO	18.34	3.48	0.44

Table 3 Correlation coefficient between the noise levels for given frequency ranges (f_1=0.125-0.25 Hz; f_2=0.5-5 Hz; f_3=2-10 Hz) and wind speed and wave height values at station LOF and HAMF for the year 2013.

	f_1	f_2	f_3
Wind LOF	0.43	0.80	0.62
Wind HAMF	0.44	0.60	0.33
Wave LOF	0.84	0.82	0.72
Wave HAMF	0.79	0.55	0.23

Table 4 Correlation coefficient (r_c) between the HAMF noise levels 2013 and wave height values at various distances (x in km) to the station.

x	r_c
50 - 100	0.74
250 - 300	0.83
450 - 500	0.86
650 - 700	0.77
850 - 900	0.73

Table 5 Spatial noise level variation of the vertical component of mainland stations for given time and frequency ranges (f_1=0.125-0.25 Hz; f_2=0.5-5 Hz; f_3=2-10 Hz). The numbers represent the average noise level of all stations minus the noise level of the individual ones in dB.

Station	January			July		
	f_1	f_2	f_3	f_1	f_2	f_3
∅ noise level	-121.82	-141.26	-146.39	-141.55	-142.95	-146.17
AKN	-4.18	1.04	2.06	0.89	0.55	-0.44
ARE0	0.95	5.25	10.14	2.27	4.81	5.39
ASK	1.81	0.81	1.94	-0.59	-1.01	2.41
BER	-0.11	-4.78	-10.64	2.32	-4.94	-9.40
BLS5	1.13	3.43	6.88	1.76	2.14	3.93
DOMB	-0.20	4.86	1.98	2.17	1.76	-2.28
HAMF	0.40	-3.48	-1.48	1.98	-1.52	-1.02
HOMB	5.12	-9.29	-15.92	-11.59	-8.23	-9.79
HYA	-0.25	-2.60	0.64	0.34	-1.61	2.07
KMY	1.26	-2.86	1.66	0.26	-4.07	-1.90
KONO	1.42	4.99	6.37	2.79	4.51	6.53
KONS	-3.59	-1.05	3.64	-13.37	-1.71	3.41
KTK1	0.93	2.84	4.51	-0.75	3.35	4.25
LOF	-0.41	-0.75	6.11	2.78	0.31	6.94
MOL	-0.48	-3.99	-9.49	0.99	-3.65	-8.90
MOR8	-1.43	3.79	5.46	1.71	3.55	4.91
NC602	-0.22	7.18	8.87	1.73	7.11	9.51
NSS	-1.83	2.32	4.40	-2.881	0.98	3.01
ODD1	1.13	2.58	2.47	-0.68	-1.95	-7.78
OSL	2.01	-3.67	-16.35	1.50	-4.33	-14.11
SKAR				3.40	5.58	6.19
SNART	-1.26	-0.31	-0.32	-3.27	1.10	0.41
STAV	1.81	-4.34	-9.88	1.39	-2.83	-5.53
STEI	-0.68	5.13	10.16	-0.34	4.37	9.83
SUE	-2.25	-2.59	2.71	3.23	-0.89	4.79
TBLU	-1.41	-4.81	-14.95	0.92	-5.17	-13.07
TRO	0.33	0.30	-0.97	1.04	1.79	0.63

Table 6 Spatial noise level variation of the vertical component of island stations for given time and frequency ranges (f_1=0.125-0.25 Hz; f_2=0.5-5 Hz; f_3=2-10 Hz). The numbers represent the average noise level of all stations minus the noise level of the individual station in dB.

Station	January			July		
	f_1	f_2	f_3	f_1	f_2	f_3
∅ noise level	-122.91	-133.15	-140.75	-139.52	-135.17	-141.78
BJO	-0.72	-5.64	-10.63	3.45	-2.81	-5.49
HOPEN	-0.31	-5.25	-9.01	-11.29	-12.49	-17.96
HSPB	8.18	5.95	5.11	2.53	5.68	6.85
JMIC	-10.93	-12.98	-18.00	-2.77	-5.92	-4.05
KBS	2.96	8.06	12.78	4.82	7.46	7.35
SPA0	0.83	9.87	19.74	3.26	8.07	13.30
